# Interplay between genes and social environment: from epigenetics to precision medicine

**DOI:** 10.1038/s41420-025-02580-z

**Published:** 2025-07-01

**Authors:** Sabrina Caporali, Simone Russo, Marcel Leist, Petra H. Wirtz, Ivano Amelio

**Affiliations:** 1 Chair for Systems Toxicology, University of Konstanz, Konstanz, Germany; 2 School of Psychology & Vision Sciences - George Davis Centre, University of Leicester, Leicester, England UK; 3 Department of in vitro Toxicology and Biomedicine, University of Konstanz, Konstanz, Germany; 4 Centre for the Advanced Study of Collective Behaviour, University of Konstanz, Konstanz, Germany; 5 Biological Work and Health Psychology, Department of Psychology, University of Konstanz, Konstanz, Germany

**Keywords:** Predictive markers, Genetics research

Natural and prolonged exposure to environmental factors substantially influences human physiology throughout life at the molecular and functional level. Gene expression, signal transduction, and cellular processes are altered by exposure, while influencing onset of several diseases such as obesity, cancer, and autoimmune disorders. In biomedicine, deconvoluting the relationship between genetic predispositions and environmental factors (GxE) remains a significant challenge, as genetics alone cannot predict an individual’s biological response to a specific environmental exposure. Cells, organs, and tissues can retain a “memory” of previous exposures, which, when combined with developmental and aging factors, create an epigenetic pattern that influences future responses [1]. Therefore, GxE interplay cannot be solely unravelled by examining genetics alone, a detailed understanding of cellular status can only be provided by considering epigenetic patterns. Based on this, we recently proposed the concept of an “epigenetic score metre,” a tool that can disentangle the relationship between genetic and environmental influences [2].

Social environment could also participate in the GxE. Major support to this postulation comes from evidence that early-life stress represents an important risk factor for physical disorders [3] as well as mental health conditions and cognitive disabilities [4]. Genetic predispositions, when combined with extrinsic influences, even from social environment, can significantly impact an individual’s mental health trajectory [5]. Here, we will provide a summary, supported by historical context and a few brief examples, to highlight how social interaction contributes within GxE interplay to psychological and mild psychiatric thought epigenetic mechanisms (Fig. 1). We will also discuss how deconstructing this interplay could have a significant impact on both society and the economy.

**Fig. 1:**
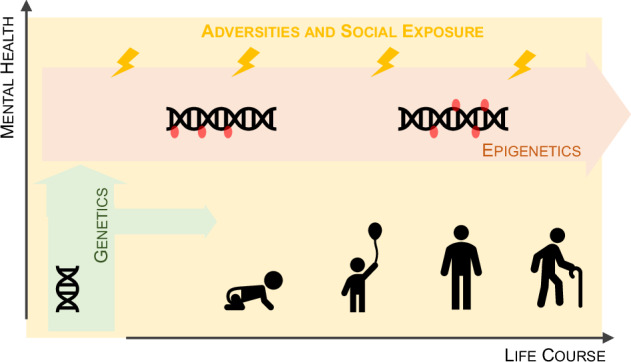
Schematic representation of the combined influence of genetic and environmental factors on mental health burden. Throughout the life course, various physical and social environmental stressors interact with individual epigenetic mechanisms. Genetic variation further modulates these responses, contributing to diverse mental health outcomes.

## Epigenetics and mental health

Epigenetics refers to the study of how environmental factors can alter gene expression without changing the underlying DNA sequence. These changes can, in turn, influence behaviour, cognition, and personality. The Diathesis/Stress model, introduced by Zubin and Spring in 1977 [6], offers a framework for understanding mental health disorders. According to this model, such conditions arise from the interaction between genetic vulnerabilities (diatheses) and environmental stressors. It suggests that individuals with a genetic predisposition to mental illness may require specific environmental triggers, such as trauma or adversity, to manifest the disorder. This model emphasizes that mental health disorders result not from genetics or environmental factors alone, but from the interplay between the two.

A twin study by Kendler and colleagues [7] highlighted that 37% of the variance in susceptibility to major depression could be attributed to genetic factors, with substantial overlap between the genetic factors influencing depression and other psychiatric disorders. Similarly, a meta-analysis by Hettema and colleagues [8] showed that between 30% and 50% of the variance in anxiety disorders could be explained by genetic predisposition, with variations depending on the specific disorder. These findings underscore the significant role of genetics in mental health. However, environmental factors also contribute to mental health conditions. A more recent adoption study revealed a significant resemblance in the prevalence of major depressive disorder between children and their adoptive families, indicating environmental transmission [7]. Even if children are not genetically related to their adoptive parents, being raised in a household where caregivers, such as step-parents, struggle with depression increases the likelihood of the child developing depression themselves. This supports the idea that environmental factors, such as the mental health of caregivers, can shape an individual’s vulnerability to mental illness.

Changes in gene expression within limbic brain have been associated to aberrant epigenetic regulations with potential causative roles in development of depression and stress-related disorders, such as post-traumatic stress disorder and various anxiety disorders [9]. Consistently, antidepressant medications may exert, at least in part, enduring therapeutic effects by being mediated through epigenetic mechanisms [9].

The environmental effects were also shown to be transmissible with mechanisms of epigenetic inheritance. In mice, chronic exposure to psychosocial stress led to a significant decrease in 5-methylcytosine levels in germ cells. Specifically, psychosocial stress appeared to alter DNA methylation in gene regulatory regions that are involved in transcriptional regulation. These findings suggest that chronic stress disrupts the male reproductive system by inducing abnormal epigenetic changes in male germ cells [10]. This indicates how chronic stress negatively affects germ cell development in the testis and that these effects can be inherited by offspring.

Behavioural traits in mammals have been linked to non-genetic transgenerational inheritance involving the germ line, with epigenetic modifications and non-coding RNAs in germ cells playing a key role [11]. These mechanisms appear similar to non-genetic transgenerational inheritance observed in parental exposure to toxicants or metabolic stress, whose offsprings display higher risk of deficits [12–14].

Consistent with epigenetic research in this area [15], development of common mental health disorders appears to be the integration of multiple influences by an interaction between genetic predispositions and environmental factors, including socioeconomic status, prior trauma, and the mental health of caregivers.

## Healthcare burden of mental health conditions

The societal impact of mental health disorders, both common and severe, is profound. Research has shown that mental health problems, including common conditions like depression and anxiety, and more severe conditions such as psychosis, place a significant burden on healthcare systems [16, 17]. This burden arises from high hospitalization rates, comorbidity with physical long-term conditions, and the need for prolonged psychological and pharmacological interventions. In 2019 alone, major depression accounted for a staggering $333.7 billion in economic costs in the United States. Moreover, mental health conditions can severely affect an individual’s ability to work, contributing to economic loss. Studies have shown that individuals with depression experience an average loss of 27.2 working days per year, further exacerbating the societal and economic costs associated with these conditions [18, 19].

These findings highlight the importance of understanding and predicting the GxE interactions that contribute to mental health disturbances. Such understanding is vital for preventing the spread of these conditions and reducing their impact on society.

The implementation of predictive strategies in public health services, such as predictive mathematical modelling based on epigenetic profiling capable of estimating an individual’s likelihood of developing specific mental health conditions, could enable targeted and effective interventions. By screening high-risk populations and offering early interventions, it would be possible to mitigate the onset and severity of mental health issues. Early intervention, particularly for patients experiencing psychosis, has been shown to reduce symptoms and minimize long-term impacts on functioning [20].

## Precision medicine in mental health treatment

Recent advances in psychiatric research have suggested that precision medicine, tailored to an individual’s unique genetic and environmental profile, could play a transformative role in mental health care. As seen in other areas of medicine, such as oncology and cardiology, precision medicine has the potential to improve outcomes by providing individualized treatments based on genetic makeup and environmental exposure. In psychiatry, this could involve personalized pharmacological interventions, accounting for both genetic vulnerabilities and environmental exposures, to optimize treatment efficacy.

Incorporating an epigenetic approach into precision medicine could offer new opportunities for mental health care. By identifying high-risk individuals early, and tailoring interventions to their specific needs, healthcare systems can provide more effective treatments, potentially improving the quality of life for patients while reducing the long-term costs associated with mental health disorders. This approach could also facilitate greater integration of affected individuals into the workforce, reducing absenteeism and improving economic outcomes.

The interaction between genetic and environmental factors plays a crucial role in the development of mental health disturbances. Epigenetics can provide a valuable framework for understanding how environmental influences shape gene expression and, consequently, mental health outcomes. As research continues to unravel these relationships, the application of predictive models and precision medicine in mental health care holds promise for early intervention and more effective treatments. By incorporating epigenetic insights into public health strategies and mental health care, it is possible to improve patient outcomes, reduce healthcare costs, and alleviate the societal burden of mental health disorders.
